# The Use of Grape By-Products as a Feed Additive Enhances the Oxidative Stability of Rabbit Meat

**DOI:** 10.3390/vetsci12020148

**Published:** 2025-02-10

**Authors:** Silvia Carta, Riccardo Chessa, Roberto Rubattu, Anna Nudda, Gianni Battacone

**Affiliations:** Dipartimento di Agraria, University of Sassari, Viale Italia, 39, 07100 Sassari, Italy; scarta2@uniss.it (S.C.); rchessa@uniss.it (R.C.); rubattu@uniss.it (R.R.); battacon@uniss.it (G.B.)

**Keywords:** oxidative stability, rabbit meat, grape by-product, fatty acids profile

## Abstract

The use of agro-industrial by-products in livestock nutrition is of increased interest to the feed industry. Some of the by-products are rich in bioactive compounds that could lead to an increase in the quality of animal meat products. This study aimed to evaluate the effects of dietary incorporation of grape pomace (GP), a by-product rich in polyphenols, on growth and on the oxidation status and the fatty acid profile of rabbit meat. Forty-eight male rabbits (*Oryctolagus cuniculus*) (aged 55 ± 3 d and ca 2.3 kg) divided into three groups of 16 were used in the trial. The rabbits were housed in pairs in wire-mesh cages (26 × 46 × 35 cm) giving n = 8 for each diet treatment. The first group was fed a basal diet (CTR) (ca 18% protein and ca 4% fat), the second was given a basal diet supplemented with 5 g GP per head per day (low-GP) and the third group was fed a basal diet supplemented with 10 g GP per head per day (high-GP). The trial lasted three weeks, and the animals were weighed weekly. The rabbits were slaughtered, and the weight and the pH of the carcasses was measured immediately after slaughter and after 24 h of cooling at 4 °C. The pH of the stomach and cecum was measured. A sample of *Longissimus Dorsi* (LD) was collected and stored at −80 °C for the analysis of proximate composition, thiobarbituric acid reactive substances (TBARs) and fatty acid profile (FA). The diet did not influence the growth or the pH of the stomach and cecum of the rabbits. The chemical composition of the meat was similar in all groups, with a mean protein of 22.3% and fat of 0.95%. The fatty acid profiles of the LD were not influenced by the diet, and the most abundant FAs were C16:0, C18:2n6 and C18:1c9. Feeding GP to the rabbits gave a linear decrease of TBARs in the meat, indicating a reduction in oxidative stress. The TBARs’ reduction was 60% in the meat of the rabbits given high-GP compared to CTR. In conclusion, GP could be used in rabbit diets without any negative influence on meat quality but with an increase in oxidation stability that could enhance meat shelf-life.

## Simple Summary

Grape pomace is a by-product rich in bioactive compounds with antioxidant and anti-inflammatory properties. The inclusion of grape pomace in the diet of rabbits (*Oryctolagus cuniculus*) improves the oxidative stability of the meat, enhancing the quality of the product. The inclusion of a small amount of this by-product in rabbit diets had no negative effect on live weight and on the average daily gain of the animals. For these reasons, it could be introduced in rabbit diets to enhance the circular economy in the agro-industrial and livestock sectors.

## 1. Introduction

Rabbit (*Oryctolagus cuniculus)* meat is an important source of high-value amino acids, vitamins and minerals [1]. It is rich in protein (22% for the *Longissimus dorsi*), lysine, sulfur-containing amino acids, threonine and valine. These amino acids play an important role in the prevention of metabolic diseases [2], in the regulation of skeletal muscle function [3] and in human and animal intestinal health [4]. Compared to other meats, rabbit meat has a higher concentration of vitamin B_12_ [5] (8.7–11.9 µg/100 g of lean edible portion). This vitamin plays an important role in human health, contributing to a reduced risk of colon cancer, cardiovascular and cognitive diseases [6]. Vitamin B_12_ cannot be synthetized by the human body and is obtained via the diet [7]. The consumption of 100 g of rabbit meat provides three times the recommended daily intake of 2 µg/day of vitamin B_12_ for a human adult [8]. Lipid concentrations vary depending upon the part of the carcass considered. For example, the leanest cut is the loin, with a lipid content of 1.8 g/100 g of meat, while the fattiest portion is the foreleg, with a lipid content of 8.8 g/100 g of meat [1]. Rabbit meat is high in unsaturated fatty acids (UFA), which account for ca 60% of the total fatty acids (FA), and more than half of these are polyunsaturated fatty acids (PUFA). This is high compared to other meats, including poultry meat [9]. Nutritional strategies, such as dietary supplementation with linseed, rapeseed or fish oil, can be used to increase meat PUFAs [10], but a high concentration of PUFAs can lead to an increased risk of oxidation and a reduction in meat shelf-life. Meat (muscle) is rich in prooxidant metals and generally poor in endogenous antioxidants, making PUFAs susceptible to oxidation [1]. Therefore, when dietary manipulation is used to increase meat PUFAs, ingredients with antioxidant properties must be included to provide a protective effect.

Grape pomace (GP) is a by-product of wine production, and it includes the skin, the pulp and the seeds. GP is rich in phenolic compounds that might have beneficial effects on the prevention of UFA oxidation and, consequently, on the quality of animal products [11]. The inclusion of GP in the diet of sheep increased the antioxidant status of the blood (increasing the FRAP and ABTS parameters) and milk (decreasing the protein carbonyls parameter) [11] and improved the milk yield and its quality [11,12]. In addition, the inclusion of this type of by-product in animal feed could improve the sustainability of the meat production chain and reduce the amount of agro-industrial waste to be disposed of. On this basis, the aim of this work was to evaluate the effect of the inclusion of GP in rabbit diets and its effect on growth performance, approximate chemical composition, fatty acid profile and on the oxidative status of the *Longissimus dorsi* (LD) muscle.

## 2. Materials and Methods

The experiment complied with animal experimentation regulations, and the animals were treated in accordance with the European Union guidelines on animal care [13]. The rabbits used in the study were reared in accordance with Legislative Decree No. 146, which transposes the European Directive [14].

### 2.1. Animals and Diet

The trial was carried out in a commercial farm located in the north of Sardinia. Forty-eight male crossbred rabbits, aged 55 ± 3 d and with an initial body weight of 2336 ± 157 g, were used in the experiment. Rabbits were housed in a controlled environment at 24 °C with ad libitum access to feed and water. The animals were housed in wire-mesh cages (dimensions 26 × 46 × 35 cm). The rabbits were randomly assigned to three treatment groups, and each experimental group consisted of 16 rabbits, with 8 replicates, and each replicate had 2 rabbits (2 rabbits/cage). The first group was fed a basal diet (CTR); a second group was fed a basal diet and a supplement of 5 g/day per head of GP (low-GP); a third group was fed a basal diet and a supplement of 10 g/day per head of GP (high-GP). The basal diet was a commercial concentrate administrated ad libitum to the animals (Table 1). The commercial concentrate contained wheat bran, sunflower seed meal, barley, alfalfa flour, beet pulp, sugar cane molasses, wheat flour, calcium carbonate, palm vegetable oil and sodium chloride. GP was supplied by a winery from the region of Sardinia in Italy. The by-product was fed once a day and the operators visually checked that each animal consumed the by-product. The trial lasted 3 weeks.

### 2.2. Measurement and Sample Collection

Rabbit body weights (BW) were recorded weekly (three times during the trial), and the average daily gain (ADG) was calculated. At the end of the experiment, the rabbits were electrically stunned and slaughtered by cutting the carotid artery. After slaughtering, the intestinal tract was removed. The so-called carcass included the head, the liver, the thoracic organs and the kidney. Carcass weight was measured immediately after the slaughter (hot carcass weight) and after 24 h of cooling at 4 °C (cold carcass weight). Carcass yields were calculated as the percentage of the hot and cold carcass weights relative to the final BW, measured immediately before slaughter. The pH was measured in the left side of the LD at 45 min and 24 h after the slaughter. The pH of the stomach and the cecum was measured 45 min after the slaughter. A temperature-compensated pH meter (Thermo Scientific (Waltham, MA, USA) 0250A0 pH/mV/relative mV/temperature meter, model 250A, Orion Research Inc., Boston, MA, USA), calibrated at regular intervals before use with pH 4 and 7 reference solutions, was used for pH measurement. A sample of the LD was dissected and stored at −80 °C for the analysis of gross composition, fatty acid profile and TBARs.

### 2.3. Laboratory Analysis

#### 2.3.1. Feed Analysis

Feed samples (commercial concentrate and GP) were analyzed for dry matter (DM, 105° for 24 h), ash and crude protein (CP) according to the methods of AOAC [15]. Feed samples were also analyzed for neutral detergent fibers (NDF), following the method developed by Mertens [16], acid detergent fibers (ADF), according to the method of the AOAC [17] and for acid detergent lignin (ADL), following Robertson and Van Soest [18]. Ether extract (EE) was measured using the Soxhlet extraction method [15]. The fatty acid content of the feed was analyzed following Correddu et al.’s protocol [19]. Briefly, 2 mL of sodium methoxide 0.5 M in methanol was added to 100 mg of powered samples and placed in a water bath at 50 °C for 10 min. The samples were then cooled at room temperature and then 3 mL of HCl/methanol (3 M), prepared with acetyl chloride and methanol, was added. The samples were placed again in the water bath in the same condition described above. Then, 1 mL of internal standard, containing methyl nonadecanoate (C19:0, Sigma Chemical Co., St. Louis, MO, USA) and 7.5 mL of K_2_CO_3_ (0.43 M), was added. The samples were centrifuged (1500× *g*, room temperature, 5 min), and the supernatant was retained in a vial for GC analysis. A 7890A GC System (Agilent Technologies, Santa Clara, CA, USA), equipped with a 7693 Autosampler (Agilent Technologies, Santa Clara, CA, USA) and a flame ionization detector (FID), were used for the determination of FAs. The carried gas used was helium (1 mL/ min flow rate), and the pressure was 28 psi. The instrument maintained an initial temperature of 45 °C for 4 min and then was increased by 13 °C/min until it reached 175 °C. This temperature was maintained for 27 min and then increased by 4 °C/min to 215 °C. The temperature of the injector and the detector was 250 °C. The split ratio was 1:80. The OpenLAB CDS GC ChemStation Upgrade software data system (Revision C.01.04, Agilent Technologies Inc., Santa Clara, CA, USA) was used to determine the area of the FAME. Following Nudda et al. [20], the peaks were identified by comparing their retention time with that of the methyl standard. The individual FA was expressed as a percentage of the total FAME. The total polyphenol concentration of the GP was determined by the Folin–Ciocalteu method as described by Kim et al. [21] and modified by Nudda et al. [22].

#### 2.3.2. Chemical Analysis of LD

LD samples were examined for proximate analysis. Approximately, 25 g of LD was used for the determination of moisture, total protein and fat content. Crude protein was determined using the method described above for the feed samples, while the fat content was determined following Folch et al.’s method [23]. Fat extraction was performed using 1 g of a lyophilized and finely ground sample. Briefly, 30 mL of chloroform:methanol (2:1) was added to 1 g of the sample. The sample was vortexed for 30 s, sonicated for 5 min and then centrifuged at 1500× *g* for 10 min at room temperature. The supernatant was filtered through a Whatman filter paper and 6 mL of 1% NaCl (*w*/*v*) was added. The sample was centrifuged again as described above. The upper part containing methanol:water was removed, and the chloroform extract was evaporated. Subsequently, the extracted lipids were methylated and analyzed by gas-chromatography as detailed above for feed samples. The peaks were identified by comparing their retention time with that of the methyl standard [24]. The individual FA was expressed as a percentage of the total FAME. Saturated fatty acids (SFA) were calculated as the sum of the individual saturated fatty acids, unsaturated fatty acids (UFA) as the sum of the individual unsaturated fatty acids, monounsaturated fatty acids (MUFA) as the sum of the individual monounsaturated fatty acids, polyunsaturated fatty acids (PUFA) as the sum of the individual polyunsaturated fatty acids, branched-chain fatty acids (BCFA) as the sum of the individual branched-chain fatty acids and odd- and branched-chain fatty acids (OBCFA) as the sum of the individual odd- and branched-chain fatty acids.

The analysis of the thiobarbituric acid reactive substances (TBARs) was performed following the method by Raharjo et al. [25]. Briefly, 10 g of samples were added with butylated hydroxytoluene (0.15%). A solution of trichloroacetic acid (TCA; 40 mL, 5% *w*/*v*) was used to homogenize the sample. Then, the sample was centrifuged at 5000× *g* for 45 min at 5 °C, filtrated and made up to the mark with TCA (5% aqueous). 2 mL of the filtrate was mixed with 2-thiobarbituric acid (TBA, 40 mM) and heated at 93 °C for 20 min to develop a rose-pink color. The absorbance was measured using a spectrophotometer at 525 nm. A solution containing TCA (2 mL, 5% *w*/*v*) and TBA (2 mL 40 mM) was used as a blank.

A standard curve (0.2–20 μM) of 1,1,3,3-tetraethoxypropane (TEP) was used to calculate the TBARs’ values that were expressed as mg of MDA per kg of dry meat. The conversion of TEP to the MDA concentration was prepared using a formula proposed by Pikul et al. [26] as

TBARs_(mg MDA/kg dry meat)_ = ((A × m × 72.063 × 10^−6^)/E) × 1000

where

A is the absorbance of the sample;
m is the slope of the calibration curve;
72.063 is the molecular weight of malondialdehyde;
E is the sample weight equivalent.

Two replicates were run per sample.

### 2.4. Statistical Analysis

Data on live body weight and ADG were analyzed using the PROC MIXED of SAS. The model included the diet and the time, their interaction as the fixed effect and the animal as the random effect.

Data on carcass weight, cold carcass weight, pH of carcasses, stomach and cecum, approximate chemical analysis, FAME and TBARs were processed using the PROC GLM procedure of SAS. The model included the group as a fixed effect.

## 3. Results

### 3.1. Animal Performance and Carcass Traits

**Figure 1:**
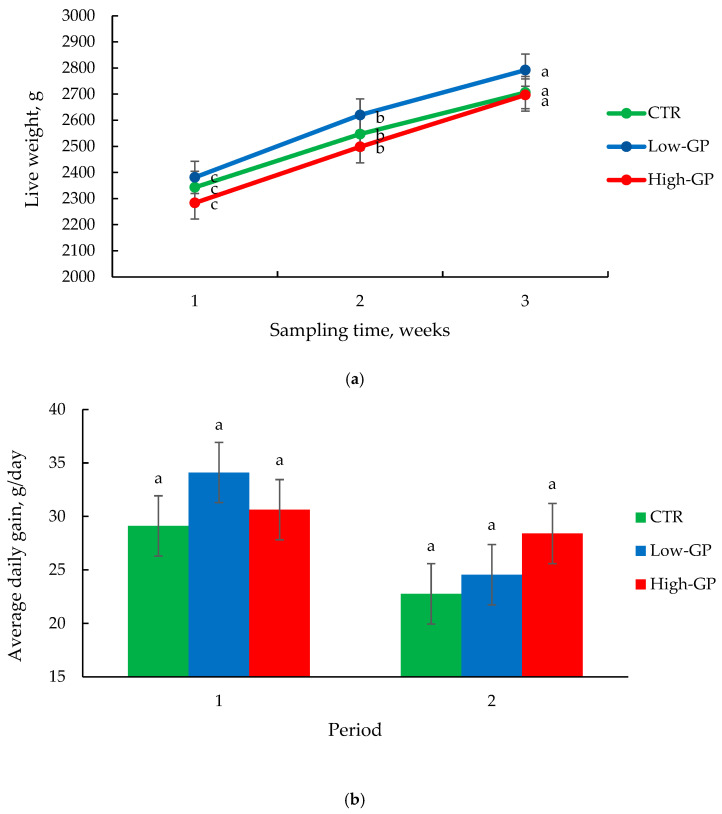
Live body weight (**a**) and average daily gain (ADG, g/d) (**b**) of rabbits of the three experimental groups during the trial. Different letters within the same group show a statistical difference (*p* < 0.05) over time. Data are presented as mean ± SE.

The performance traits of the rabbits fed a diet supplemented with GP are shown in Figure 1. The live weight and the ADG were not influenced by the diet (*p* > 0.05), but they were influenced by the time factor (*p* < 0.001, Figure 1). The overall mean of final body weight was 2732 g. The carcasses’ traits are shown in Table 2. Hot and cold carcasses’ weights were not affected by the treatment (*p* > 0.05). The pH of the carcass, the stomach and the cecum of animals fed GP did not differ from that of the CTR (*p* > 0.05).

### 3.2. Proximate Composition and Fatty Acid Profile

Proximate analysis and the fatty acid profile of the LD of rabbits fed GP are shown in Table 3.

The diet did not influence the chemical composition of the LD (*p* > 0.05). The fat concentration of the three experimental groups was 0.91, 1.00 and 0.93% for CTR, low-GP and high-GP, respectively. The protein concentration did not significantly differ among groups, and the mean values were 22.6, 22.2 and 22.1% for CTR, low-GP and high-GP, respectively.

The fatty acid profile of rabbit meat is shown in Table 3. The two doses of GP included in the diet did not affect the fatty acid profile of the LD (*p* > 0.05). The most abundant fatty acid in rabbit meat was the palmitic acid (C16:0), followed by linoleic acid (C18:2n6) and oleic acid (C18:1c9) representing 76% of the total FA. The UFA represented 60% of the total FA, provided for the 26% of the MUFA and the 33% of the PUFA, while the SFA represented 40% of the total FA.

### 3.3. TBARs

**Figure 2:**
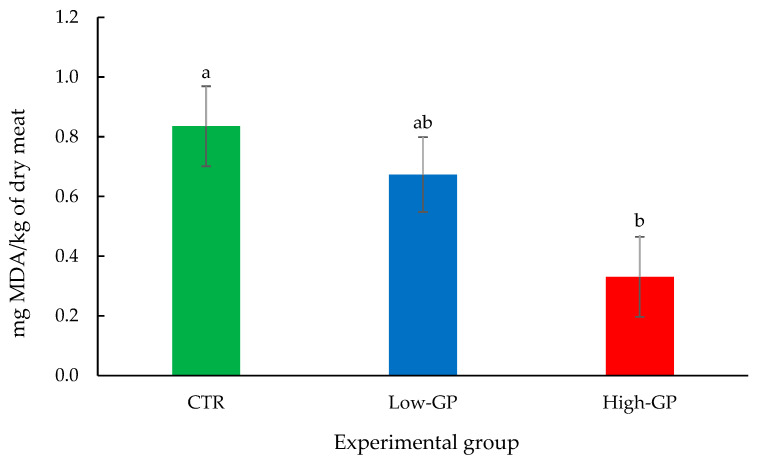
Effect of the two doses of grape pomace on TBARs in LD muscle. Data are presented as mean ± SE. Different letters indicate significant differences between groups’ mean.

The results of the TBARs’ analysis are shown in Figure 2. GP supplementation resulted in a linear decrease in mg of MDA per kg of dry meat. The values of mg of MDA per kg of dry meat were 0.83, 0.67 and 0.33 for the CTR, low-GP and high-GP groups, respectively.

## 4. Discussion

The inclusion of GP in the diet of fattening rabbits had no negative effect on the growth of the animals, as evidenced by the live body weight and the ADG. These results were in agreement with those obtained by Bouzaida et al. [27] who administered a diet supplemented with 20% of GP to fattening commercial rabbits. In the same study, the authors found a reduction in carcass yield in animals fed grape by-products. These results are in contrast with those found in our study, where hot and cold carcass weight and carcass yields were not affected by the two doses of GP. These discrepancies are probably due to the different percentages of GP in the diet of rabbits, which were higher in the study by Bouzaida et al. [27] than in our study. The pH of the LD, both at 45 min and at 24 h, did not differ between groups. When growth rate is not affected by dietary treatments, a little or no effect on pH would be expected [28]. However, the pH at 45 min and at 24 h after slaughter was within the range for the same cut [27,29]. The pH of the stomach and the pH of the cecum did not differ between treatments, suggesting that the tested doses of this by-product did not affect the activity of gut microflora.

The inclusion of GP in the rabbits’ diet did not affect the chemical composition of the LD. The percentage of fat found in the LD was low but in agreement with the results of other studies that considered the same cut [30]. However, Bouzaida et al. [27] found an increase in fat concentration in the meat of rabbits supplemented with 20% of GP. The dose used in our study was probably not sufficient to modify the fat concentration in the LD. Similarly, the protein concentration was not significantly different between groups. The GP supplementation did not influence the fatty acid profile of the LD. However, Bouzaida et al. [27] observed that a diet containing 20% of GP induced an increase in the concentration of PUFA and a decrease in SFA in the LD of rabbits, probably due to the higher dose of the by-product. This has been observed in other monogastric animals as piglets, where the inclusion of 9% of GP in the diet resulted in an increase in linolenic acid (C18:3 n-3, LNA), eicosapentaenoic acid (C20:5 n-3, EPA), docosahexaenoic acid (C22:6 n-3, DHA), PUFA and PUFA n-3 [31,32]. The fact that linoleic acid was the most abundant fatty acid in the basal diet and in the GP probably explains the lack of differences in the FA profile between the CTR and GP groups. The concentration of arachidonic acid (C20:4 n-6, ARA) was high in the three groups, regardless of the GP supplementation. In fact, ARA can be synthesized from linoleic acid as a result of elongation and desaturation processes [33,34,35].

The results of our study highlighted an increase in the oxidative stability of meat derived from rabbits fed with the GP. Bennato et al. [36] found that the GP intake was effective in protecting the lipid from the peroxidation in chicken meat. On the contrary, Bouzaida et al. [27] found no effect on TBARs in meat from rabbits fed with GP, even though the doses used were higher than those used in our study. The differences could be due to several factors, such as the different proximate composition of the LD, the different storage method of the sample or the processes to which it was subjected. In addition, the susceptibility of lipids to peroxidation depends on the proportion of PUFAs, on the amount of reactive oxygen the species produced and on the amount of antioxidant endogenous that arise from diet [37].

Other studies, carried out on rabbits but using a different source of polyphenols in the diet, have shown a reduction in TBARS in meat, demonstrating the ability of polyphenols to increase the oxidative stability of the product. For example, Rossi et al. [38] found an enhancement of oxidative stability when rabbits were fed 0.3% of brown seaweed and 0.6% of a polyphenol mixture, while Liu et al. [39] showed a decrease of TBARS of meat from rabbits fed 0.1% chestnut tannins. The polyphenols are responsible for the increase in the oxidative stability, and they lead to a delay in lipid oxidation in the meat, as reported by other authors [40,41,42]. In fact, polyphenols have a high antioxidant activity due to the presence of hydroxyl substituents and their aromatic structure, which allows them to scavenge the free radicals [42].

## 5. Conclusions

In conclusion, 5 and 10 g/d of GP can be used in the diet of rabbits during the fattening period without negative effects on growth performance and meat quality. The inclusion of this by-product in the diet of these animals could be a good nutritional strategy to improve the oxidative stability of the meat, as demonstrated by the reduction in MDA per kg of meat. Unexpectedly, the use of the GP in the diet did not modify the FA profile of the meat, probably because the doses were not sufficient to highlight any difference. However, this by-product could be usefully employed in rabbit diets to improve the oxidative stability of the meat and enhance the sustainability of the livestock supply chain.

## Figures and Tables

**Table 1:** Chemical composition and fatty acids’ profile of the commercial concentrate and the by-product administered to the rabbits.

Items	Concentrate	Grape Pomace
Chemical composition (% of DM unless otherwise noted)		
DM, %	88.16	25.35
NDF	32.84	50.97
ADF	19.04	45.09
ADL	4.84	32.10
CP	17.76	13.23
Ash	7.81	7.00
EE	3.70	5.05
Total polyphenols, g GAE/100 of DM	-	1.48
FAME, g/100 g of total FAME		
C14:0	0.23	0.29
C16:0	17.44	14.06
C18:0	3.25	4.02
C18:1 cis-9	25.90	17.18
C18:1 cis-11	1.13	1.02
C18:2 n-6 (LA)	44.27	55.35
C18:3 n-6	0.08	0.00
C18:3 n-3 (LNA)	4.49	2.94
C22:0	0.44	0.96
C20:4 n-6 (ARA)	0.14	0.26
C24:0	0.55	0.45
C22:5 n-3 (DPA)	0.13	0.66
Groups of FA (g/100 g of total FAME)		
SCFA	0.00	0.08
MCFA	18.15	15.32
LCFA	81.85	84.60
SFA	22.54	21.09
MUFA	28.08	19.28
PUFA	49.38	59.63
UFA	77.46	78.91
PUFA n-6	44.71	55.81
PUFA n-3	4.62	3.68
n-6/n-3	9.67	15.16
n-3/n-6	0.10	0.07

DM = dry matter; CP = crude protein; EE = ether extract; NDF = neutral detergent fiber; ADF = acid detergent fiber; ADL = acid detergent lignin; LA = linoleic acid; LNA = linolenic acid; ARA = arachidonic acid; DPA = docosapentaenoic acid; SCFA = short-chain fatty acids, sum of the individual fatty acids from C4:0 to C10:0; MCFA = medium-chain fatty acids, sum of the individual fatty acids from C11:0 to C17:0; LCFA = long-chain fatty acids, sum of the individual fatty acids from C18:0 to DHA; SFA = sum of the individual saturated fatty acids; MUFA = sum of the individual monounsaturated fatty acids; PUFA = sum of the individual polyunsaturated fatty acids; UFA = sum of the individual unsaturated fatty acids; and PUFA n-3 and PUFA n-6 = sum of individual n-3 and n-6 fatty acids, respectively.

**Table 2:** Effect of the two doses of grape pomace on weight and yield of the carcass and on the pH of the *Longissimus dorsis*, stomach and cecum.

	Diet			SEM	*p*-Value
	CTR	Low-GP	High-GP		
Slaughter weight, g	2706.25	2791.88	2696.88	37.25	0.84
Hot Carcass weight, g	1604.38	1628.13	1588.13	26.35	0.84
Cold carcass weight, g	1563.13	1600.63	1558.75	25.40	0.78
Hot carcass yield, %	59.23	58.31	58.91	0.53	0.79
Cold carcass yield, %	57.74	57.32	57.81	0.52	0.93
pH LD, 45 min	6.51	6.38	6.41	0.05	0.57
pH LD, 24 h	5.74	5.66	5.68	0.03	0.59
pH stomach	1.61	1.61	1.62	0.04	0.99
pH cecum	5.94	5.85	5.88	0.04	0.74

CTR = animals fed the basal diet; low-GP = animals fed the basal diet and a supplementation of 5 g/d of grape pomace; high-GP = animals fed the basal diet and a supplementation of 10 g/d of grape pomace. LD = *Longissimus Dorsi*.

**Table 3:** Effect of the two doses of grape pomace on proximate chemical analysis and on the fatty acid profile of the *Longissimus dorsi*.

Items	Diet			SEM	*p*-Value
	CTR	Low-GP	High-GP		
Moisture, %	75.33	75.45	75.65	0.133	0.63
Protein, %	22.62	22.23	22.11	0.130	0.25
Fat, %	0.91	0.99	0.93	0.037	0.67
Individual FA (g/100 g of FA)					
C14:0	1.17	1.07	1.23	0.098	0.80
isoC15:0	0.03	0.04	0.04	0.002	0.88
anteisoC15:0	0.06	0.06	0.06	0.003	0.98
C15:0	0.51	0.51	0.50	0.007	0.67
isoC16:0	0.13	0.13	0.13	0.007	0.99
C16:0	29.06	28.64	28.78	0.231	0.76
isoC17:0	0.02	0.02	0.02	0.001	0.04
C16:1 cis-7	0.35	0.37	0.38	0.015	0.74
anteisoC17:0	0.05	0.04	0.05	0.003	0.42
C16:1 cis-9	1.20	1.38	1.53	0.198	0.81
C17:0	0.63	0.61	0.60	0.010	0.38
C18:0	9.07	8.90	8.75	0.159	0.74
C18:1 cis-9	23.24	22.74	23.04	0.476	0.92
C18:1 cis-11	1.45	1.58	1.46	0.031	0.19
C18:2 n-6 (LA)	23.80	24.55	24.48	0.255	0.44
C20:0	0.09	0.09	0.09	0.004	0.69
C18:3 n-6	0.07	0.08	0.07	0.002	0.49
C20:1 cis-11	0.18	0.20	0.19	0.007	0.67
C18:3 n-3 (LNA)	0.94	0.93	1.00	0.073	0.92
C20:2 n-6	0.30	0.33	0.35	0.015	0.42
C20:3 n-6	0.60	0.63	0.62	0.032	0.94
C20:4 n-6 (ARA)	5.92	5.96	5.59	0.423	0.93
C20:5 n-3 (EPA)	0.26	0.29	0.27	0.016	0.77
C22:5 n-3 (DPA)	0.71	0.71	0.64	0.052	0.84
C22:6 n-3 (DHA)	0.11	0.12	0.11	0.010	0.91
Groups of FA (g/100 g of FA)					
MCFA	33.20	32.85	33.31	0.327	0.85
LCFA	66.80	67.15	66.69	0.327	0.85
SFA	40.81	40.10	40.24	0.272	0.55
MUFA	26.42	26.26	26.60	0.621	0.98
PUFA	32.76	33.64	33.16	0.586	0.84
UFA	59.19	59.90	59.76	0.272	0.55
OCFA	1.14	1.12	1.09	0.015	0.46
BCFA	0.29	0.28	0.29	0.013	0.99
OBCFA	1.43	1.40	1.38	0.021	0.70
PUFA n-6	30.70	31.55	31.11	0.578	0.85
PUFA n-3	2.02	2.04	2.02	0.033	0.96
n-6/n-3	15.27	15.50	15.47	0.333	0.96
n-3/n-6	0.07	0.07	0.07	0.001	0.98
Nutritional indices					
AI	0.57	0.55	0.57	0.009	0.65
TI	0.87	0.85	0.86	0.012	0.68
h/H	1.89	1.94	1.91	0.024	0.72

CTR = animals fed the basal diet; low-GP = animals fed the basal diet and a supplementation of 5 g/d of grape pomace; high-GP = animals fed the basal diet and a supplementation of 10 g/d of grape pomace. LA = linoleic acid; LNA = linolenic acid; ARA = arachidonic acid; EPA = eicosapentaenoic acid; DPA = docosapentaenoic acid; DHA = docosahexaenoic acid; MCFA = medium-chain fatty acids, sum of the individual fatty acids from C11:0 to C17:0; LCFA = long-chain fatty acids, sum of the individual fatty acids from C18:0 to DHA; SFA = saturated fatty acids, sum of the individual saturated fatty acids; MUFA = monounsaturated fatty acids, sum of the individual monounsaturated fatty acids; BCFA = branched-chain fatty acids, sum of individual branched-chain fatty acids; OBCFA, the odd- and branched-chain fatty acids, sum of individual odd- and branched-chain fatty acids; PUFA = polyunsaturated fatty acids, sum of the individual polyunsaturated fatty acids; UFA = unsaturated fatty acids, sum of the individual unsaturated fatty acids; PUFA n-3 and PUFA n-6 = sum of individual n-3 and n-6 fatty acids, respectively; AI = aterogenic index calculated as AI = [12:0 + (4 × 14:0) + 16:0]/[(PUFA) + (MUFA)]; TI = trombogenic index calculated as TI = (14:0 + 16:0)/ [(0.5 × MUFA) + (0.5 × n-6) + (3 × n-3) + (n-3:n-6)]; h/H = hypocholesterolemic to hypercholesterolemic ratio calculated as h/H = [(sum of c9–18:1, c11–18:1, 18:2n-6, 18:3n-6, 18:3n-3, 20:3n-6, 20:4n-6, 20:5n-3, 22:4n-6, 22:5n-3 and 22:6n-3)/(14:0 + 16:0)].

## Data Availability

All data generated during the study are available from the corresponding author upon reasonable request.
